# Infecting minds: socio-contextual drivers of vaccine perceptions and attitudes among young and older adults living in urban and rural areas in KwaZulu-Natal, South Africa

**DOI:** 10.1186/s12889-025-22181-w

**Published:** 2025-03-21

**Authors:** Kingsley Stephen Orievulu, Sally Frampton, Philippa C. Matthews, Nondumiso Mpanza, Thabisile Mjilo, Samukelisiwe Nxumalo, Joshua Hordern, Janet Seeley

**Affiliations:** 1 Africa Health Research Institute, KwaZulu-Natal, Durban, South Africa; 2 Centre for Africa China Studies, University of Johannesburg, Johannesburg, South Africa; 3 School of Nursing and Public Health, College of Health Sciences, University of KwaZulu-Natal, Durban, South Africa; 4 Division of Infection and Immunity, University College London, Gower Street, London, WC1E 6BT UK; 5 Faculty of History, University of Oxford, Oxford, UK; 6 The Francis Crick Institute, 1 Midland Road, London, NW1 1AT UK; 7 Department of Infectious Diseases, University College London Hospital, Euston Road, London, NW1 2BU UK; 8 Faculty of Theology and Religion, Harris Manchester College, University of Oxford, Oxford, UK; 9 Global Health and Development Department, London School of Hygiene and Tropical Medicine, London, UK

**Keywords:** COVID-19, Medical pluralism, Public engagement, Vaccine confidence, Vaccine hesitancy, South Africa

## Abstract

**Background:**

We investigated how social and contextual factors, including a pandemic, shape vaccine perceptions and attitudes among people living in KwaZulu-Natal in South Africa. We assessed how participants’ views, acceptance, and uptake of vaccines for a range of infectious diseases, may be influenced by experiences and events linked to the COVID-19 pandemic.

**Methods:**

We conducted 30 in-depth face-to-face and telephonic interviews with participants living in diverse rural and urban communities in two districts within KwaZulu-Natal. Participants were adults (≥ 18 years) consisting of ordinary citizens, traditional healers, and nurses. We combined non-representative convenience, snowballing and purposeful sampling techniques to recruit participants. Data collection was conducted in IsiZulu, and we used both inductive and deductive thematic analysis approaches to identify key themes linked to participants’ perceptions and attitudes towards vaccines.

**Findings:**

Our study participants were mostly those who had accepted vaccination. The main reasons given for vaccine uptake included understanding the importance of vaccines for disease prevention and survival, and securing the health of family members, the fear of death, government campaigns, vaccine mandates and penalties. Older participants (≥ 40 years) demonstrated more positive attitudes towards vaccines. Most participants downplayed the role of culture and religion in attitudes towards vaccines. However, some of the drivers of vaccine hesitancy were having an ancestral calling, medical pluralism, or local myths around the treatment of infections such as influenza and mumps, and a perceived depopulation agenda couched in mistrust and the use of incentives and penalties to force people to accept COVID-19 vaccines.

**Conclusion:**

Exploring what shapes attitudes towards vaccines in communities provides opportunities to understand the reasoning behind how people make decisions about whether to take a vaccine in different geographical and cultural spaces. The exploration of contexts, exposures and circumstances provide insights into perceptions and behaviour. Deeper engagement with local communities is crucial to develop evidence that can inform vaccine interventions. Assumptions about how culture and religion affect vaccine hesitancy or acceptance should be avoided in the process of developing such evidence.

## Background

In 2019, the World Health Organisation (WHO) listed vaccine hesitancy as one of the top ten threats to global health [1]. Within the chaos unleashed by the global COVID-19 pandemic, vaccination was emphasised as a fundamental exit strategy. But vaccine hesitancy also re-emerged as a challenge at the forefront of the public health agenda. The phenomenon - and the concept - of vaccine hesitancy is complex. It encapsulates beliefs and behaviour, underpinned by historical events, diverse cultural, political, and religious perspectives, and media representation. It may also embrace issues such as accessibility to vaccines and limited resources for communication strategies [2].

Though mainstreamed in global health discourse, vaccine hesitancy is often misrepresented in the popular media. It is not usually a case of outright rejection of vaccines. Rather, hesitancy tends towards a spectrum. It also points to deeper issues and questions about how individuals or groups engage with immunisation against the backdrop of vaccines’ perceived origin(s), efficacy, risks, and what they may connote or represent, etc [3–8]. We explore vaccine hesitancy in this paper– conversant with this debate– to demonstrate the complexity, nuances, and non-linearity of vaccine perception and engagement.

In 2014, the WHO working group on vaccine hesitancy acknowledged the ‘complexity’ of hesitancy, and the many gaps in current knowledge and best practices [9]. The group highlighted the pertinence of research within high, middle and low-income countries due to the context-specificity that characterises the phenomenon across different regions of the world. Such investigations are useful to understand ‘the scope, scale and reasons underlying vaccine hesitancy to inform appropriate responses’ [9]. While the ‘anti-vax’ lobby is relatively well recognised in the Global North, the extent and nature of vaccine hesitancy among populations in Africa has been less well-described. There is also insufficient empirical work within Africa on this topic [2]. The roll-out of SARS-CoV-2 vaccines increased awareness and engagement on the subject, but adequate contextual studies remain crucial. Notable examples include studies of the scepticism surrounding the polio vaccine in Nigeria and rumours about sterilisation often attached to public health interventions in Africa [10–14]. In addition, South Africa has witnessed a significant amount of hesitancy to childhood vaccination as well as some adult vaccinations including for influenza [15]. However, there remains an insufficient understanding of the depth of this issue and the extent to which the COVID-19 pandemic may have shaped it [16]. Thus, further granular, contextual studies in the African context remain crucial.

We therefore set out to investigate beliefs, perceptions, and behaviours in people living in the KwaZulu-Natal (KZN) province of South Africa, taking into consideration public health history in the area, alongside the religious and cultural backdrop of vaccine uptake. This is not only of relevance to vaccines to prevent COVID-19 but includes an exploration of the extent to which the pandemic affected confidence (or hesitancy) in other vaccines. We also investigated differences in how people perceived incentives or penalties surrounding vaccine uptake, and how access to (mis)information impacted their awareness of, and engagement with, vaccines. These factors have implications for health care policy and practice in South Africa pertaining to deeper public engagement and awareness creation around vaccine-related interventions.

## Historical context: vaccines and medical pluralism in South Africa

Historian Karen Flint has argued that the backdrop to current iterations of vaccine engagement in Africa must be understood in relation to colonial and postcolonial histories of the continent [17]. As a society, South Africa has a deep history of contests between ‘traditional’ African treatment and western medicine– hereafter ‘biomedicine’ [18–21]. This contest dates back to colonial administrations that tried to abrogate attempts by African ‘healers’ to mainstream their practices as medicine [17, 19–22]. This is against a backdrop where the practice of African medicine was popular among the local populace especially in Zululand and the Natal, and where local healers (*Inyanga* and *Izangoma)* were powerful, given people’s preference for them to accommodate multiple needs, including treating ill-health, managing spiritual needs, and settling disputes [22]. Their role(s) often transcended (bodily) treatment to the psychosocial and political realms [23–25]. This points to how deeply ingrained the pluralistic approach to health and illness is among South Africans [26], similar to other African populations [27].

Despite its roots within non-western medical traditions of inoculation, vaccination quickly became emblematic of biomedicine in the late nineteenth century. Smallpox vaccination was mandated by some colonial authorities in Africa as early as 1894. However, objections sprung up frequently in response to programs of vaccination and the often humiliating and invasive measures they entailed for those subjugated to colonial rule. KwaZulu-Natal was no stranger to this. The annual public health report for Durban in 1925 is just one of several reports from that era which recorded that ‘the prejudice against vaccination is still much in evidence’ [28, 29]. The wording suggests long-standing discomfort with vaccines among communities that had been labelled ‘native’ by British authorities in KZN. Reports for the Durban area later in the century, in the 1970s, suggest improved vaccination rates and more culturally nuanced immunization campaigns [30]. However, public health authorities continued to oppose the use of local healing practices. The 1928 Medical, Dental and Pharmacy Act sought to try and phase out *Inyanga* herbalist practitioners who were accused by Durban public health officers at the time of practicing necromancy as well as being ‘acutely antagonistic’ to western interventions. This did little to stop people seeking their services, which have remained consistently popular [31]. But it laid the ground for sharp divisions between biomedical and ‘traditional’ practices in South Africa in the late twentieth century, and which, according to South African anthropologist Harriet Ngubane, led to ‘the habitual disposition of the Western-type health agencies to look down on practically all indigenous methods of healing’ [32]. Legal recognition and regulation of traditional healers in South Africa would not be enshrined until 2007 [17, 22, 33].

## Methods

### Study setting

This paper is an outcome of an interdisciplinary research project, titled ‘Infecting Minds: the Past, Present and Future of vaccine hesitancy in South Africa’, implemented at the Africa Health Research Institute (AHRI), KZN between December 2021 and December 2022. AHRI is an independent, transdisciplinary scientific research institute with two campuses: AHRI-Durban and AHRI-Somkhele [34]. Our team in both sites collected qualitative data from participants residing in the diverse urban and rural communities.

The eThekwini metropolitan area of Durban is the economic hub of the KZN province with a young and diverse population [35]. Somkhele on the other hand is within a district in northern KZN, which struggles with high prevalence of poverty, unemployment, and HIV [36–38].

### Study population and recruitment strategy

The South African Government instituted several regulations pertaining to activities during the COVID-19 pandemic, including lockdown phases, based on government-determined disease severity and the population’s exposure and vulnerability levels [39–42]. The consequence for our study was that due to COVID-19-related challenges and regulations, our sampling strategy entailed a combination of non-representative convenience, snowball and purposive sampling techniques [43]. Suri, citing Patton (2002), shows this approach to be useful in reducing the extent of bias linked to any single one of these approaches– especially those characteristic of convenience sampling [43].

We recruited study participants aged 18 years and above from the Durban and Somkhele areas and aimed for gender balance in the recruitment. Initial recruitment from both study sites was based on participants’ availability and willingness to participate. In Somkhele, we collaborated with the Community Advisory Board (CAB) and the Public Engagement Department (PED) at AHRI to identify and recruit potential participants. We did this by presenting the study during the monthly CAB meeting usually facilitated by the AHRI PED, inviting members of the CAB to disseminate the information about study in their communities, and working with members of the PED to obtain the contact details of community members who wanted to participate. In Durban, the PED engaged a non-profit organisation (NPO) which works with young people and introduced the study during one of their sessions. The study recruitment (factsheet) document was also shared with members of the NPO network. Individuals who indicated an interest in participating reached out to the PED personnel via WhatsApp. We then collated the contact details of all potential participants and our trained social science research assistants (SSRAs) contacted them to officially recruit them for the study.

We also recruited a few participants based on recommendations or referral made by earlier participants– usually due to refusals from those we contacted or their failure to respond to our attempts at telephonic contacts. Finally, another group of participants– nurses and peer navigators– were proactively identified and recruited as key informants based on their positionality within the health system. Their recruitment was purposive and strategic because they directly engage people visiting health facilities for routine immunisation/vaccination; they are also part of institutional/government-led vaccination initiatives. We recruited them through the assistance of the Clinical Research Department at AHRI which works closely with the health facilities under the Department of Health in the district.

Since our data collection took place during COVID-19 restrictions (2021–2022), we regularly reviewed and revised the data collection approach in line with lockdown requirements. Bearing in mind the limitation(s) of our sample size and representation, our aim was not to generalise our finding(s) across populations [43–45]. Instead, it was to describe and analyse the issues highlighted by participants and triangulate them against existing literature and common knowledge on perceptions of, and attitudes to, vaccines.

### Data collection and analysis

Between 17 May to 21 October 2022, we conducted face-to-face semi-structured in-depth interviews (IDIs) and telephonic in-depth interviews (TIDIs) with 30 adults (18 years and above) in both locations. The IDIs were conducted at the participant’s residence– or preferred location– and the AHRI-Durban meeting rooms, while TIDIs were conducted at the AHRI call centre in Somkhele. These interviews were all audio-recorded.

The IDIs and TIDIs were conducted in the local IsiZulu language using a topic guide after obtaining informed consent from the participants. The interviews were transcribed verbatim and translated into English. The transcripts were quality checked by an experienced Social Science Core data quality coordinating team before being imported into ***NVivo 12 Pro*** for coding. NM and KO commenced with open coding, identifying emerging themes, and SF, PM, JH and JS provided guidance on the structure and synthesis of the data for reporting.

We conducted a thematic analysis of the emergent data from the transcripts and field summaries [46–48]. Thematic analysis allows for flexibility, and can be used for small and large samples drawn from interviews, and other sources of qualitative data [47, 48]. This flexibility also enabled us to combine both deductive and inductive analysis processes for the study. Mainly, we had a set of thematic guiding questions:

- What factors are driving vaccines uptake or vaccine hesitancy in KZN?
- How are people influenced by their tradition, culture, religious beliefs, politics, and social media in the way they see/perceive vaccines?
- How has the COVID-19 vaccine, if at all, affected how people feel about or trust other already existing vaccines?
- How do people respond to incentives (or encouragements) to vaccinate, or restrictions if they don’t vaccinate?
- How much do people know and think about public awareness or education campaigns for vaccines in the rural and urban areas?

We initially developed codes inductively, during the open coding process. These were revised by KO and NM, and later matched with, and subsumed within, the larger framework organised around the above thematic questions.

We focused mainly on identifying dominant themes around participants’ perceptions of and attitudes towards vaccines, with attention given to the factors (or drivers) identified by these participants about their positive or negative viewpoints about vaccines. Participants were informed that the study’s focus was on historical, sociopolitical, cultural, religious and other contextual factors that influence how people perceive and engage vaccines and vaccination programmes in their society. They were also expressly informed and reminded that the study was not a study about vaccines to protect against COVID-19, although perspectives on the subject were not excluded. The interviews lasted between 45 minutes to one and half hours, covering the topic areas highlighted above with probes, culminating in about sixteen questions in all.

### Ethics

This study was approved by the Biomedical Research Ethics Committee (BREC) of the University of KwaZulu-Natal, South Africa (BREC Ref: BREC/00003409/2021), and the Oxford Tropical Research Ethics Committee (OxTREC Reference: 520 − 21). Study participants received a full explanation of the study before consent was requested. We requested both verbal and written (signed) consent from face-to-face participants. For the TIDI participants, we obtained verbal consent, electronically, and informed them that we will sign on their behalf according to the standard of procedure during the COVID-19 pandemic. Participants were reimbursed the equivalent of about US$2.5 (R50) airtime for their time. Participants who travelled to the interview location were reimbursed their full transportation fare. Only participants who expressly consented to participate in the study were interviewed for this study.

## Findings

We interviewed 30 participants across the two settings (Table 1). The initial participants in Somkhele were aged between 40 and 73 years (*n* = 11) and the later participants aged between 27 and 83 years (*n* = 9). In Durban, participants were younger adults, aged between 20 and 30 years (*n* = 10).

**Table 1:** Study participants’ characteristics

Study ID	Age Range (years)*	Reported Vaccination status– Any (V/NV)	Reported Education level	Reported Religion	Reported Religious Group
S1	40s–50s	V	T	Christian	NR
S2	50s–60s	NR	HS	Christian	NR
S3	60s–70s	V	NR	Christian	RCM
S4	50s–60s	V	HS	Christian	NR
S5	40s–50s	V	DCHS	NR	NR
S6	60s–70s	V	DCHS	Christian	NBC
S7	70s–80s	NV	T	NR	NR
S8	50s–60s	V	BM	Christian	Lutheran
S9	40s–50s	V	HS	Christian	NR
S10	60s–70s	V	BM	Christian	NBC
S11	50s–60s	V	BM	Christian	Zion
S12	30s–40s	V	M	Christian	Zion
S13	30s–40s	V	M	Christian	Zion
S14	20s–30s	V	M	Christian	Zion
S15	30s–40s	V	NR	Christian	Church of Jubilee Ministries
S16	20s–30s	V	HS	Christian	NR
S17	40s–50s	NR	T	Christian	Shembe
S18	20s–30s	NR	CT	Christian / Tradition	Zion
S19	30s–40s	NR	CT	Christian	Zion
S20	80s–90s	NR	NR	Christian	Lutheran
D21	20s–30s	NR	T	Christian	Lutheran
D22	20s–30s	NV (COVID-19)	T	Atheist	NR
D23	20s–30s	V	T	Christian	NBC
D24	20s–30s	V	HS	Christian	Zion
D25	30s–40s	V	T	Christian	Zion
D26	20s–30s	V	DCHS	Christian	Zion
D27	30s–40s	V (Anti-penalties)	NR	NR	NR
D28	30s–40s	NR	CT	Christian	NBC
D29	20s–30s	V	HS	Christian	Zion
D30	20s–30s	V; (NV -COVID-19)	T	Christian	Zion

N/B: S-Somkhele; D-Durban; BM-Below Matric; HS-High School; T-Tertiary; CT-Certification Training (like nursing); DCHS -Did not complete High School; RCM-Roman Catholic; V-Vaccinated; NV-Not Vaccinated; NR-Nothing Reported; NBC-Nazareth Baptist Church; *Estimated age

We identified three major thematic areas around vaccine perceptions and attitudes among our study participants: a sense of vaccination popularity, a sense of hesitancy, and the impact of COVID-19 on participants’ overall vaccine outlook.

## Popularity of vaccines & vaccination

At the Somkhele site, only one participant demonstrated a strong negative perspective about vaccines (Table 1), especially on SARS-CoV-2 vaccines; reporting a refusal to be vaccinated, while most of the other participants expressed positive views. In Durban, the balance was different, with only about half the participants reporting a positive attitude towards vaccines and vaccination.

Overall, there was no apparent distinction between views expressed by men and women. However, more of the younger participants (30-years and younger)– mainly from Durban– argued against vaccination than older participants.

More than half of the participants were COVID-19 vaccinated or showed a willingness to be vaccinated based on their largely positive sentiments about vaccination in general. Three of these participants emphasised their understanding of the role vaccines play in safeguarding people’s health especially during novel infectious disease outbreaks or respiratory illness, recognised to be associated with changing seasons. In their responses, these participants demonstrated moderate to high levels of confidence in the government through the Department of Health vaccination schedule which, as a participant (20–30 years old) from Durban noted, had helped him because he went to ‘Clicks [pharmacy]… for the flu vaccine and… did not have flu even after the season changed…’ Others emphasised immunisation programmes, especially those earmarked for pregnant women in line with the 2016 vaccine guidelines, including Tetanus Toxoid (TT) or Tetanus and Diphtheria (Td) [49]. A female participant (40–50 years old) from Somkhele, talking about maternal vaccines, noted:

I had a good feeling about vaccination taken when pregnant because you were informed that it helps the baby on the inside even when outside it will be alive and healthy; even if you [have] infections, the child is protected through this vaccination [and it] will help you before it gets through the child; if you didn’t take this vaccination your child might have a possibility to be infected.

Some of the reasons highlighted for their perceived positive attitudes towards vaccines and vaccine uptake (at individual levels) among all age groups and sexes included understanding the importance of vaccines for disease prevention and control, securing the health and survival of family members, addressing fears of dying, and responses to government campaigns, mandates, incentives, and penalties.

### Vaccine significance: disease prevention and health security

Participants who supported vaccination, defined vaccines as a tool for disease prevention. A man (20–30 years old) from Durban commented that ‘… it can be immunisation for children to prevent certain diseases…’. A man from Durban (20–30 years old) observed that this prevention is akin to possessing a magical protective instrument– similar to a local witchcraft amulet (‘*Uvutha*’)– which helps to protect an individual from harm. So, he continues, ‘… the vaccine is something like that [‘*Uvutha’*], which means I will infect you with this dangerous disease. Being infected with a dangerous disease is mixed for the body to get used to this thing and then be able to fight it.’

In these attempts to define vaccines, participants reflected their belief that vaccination helps them and can secure their families, accompanied by a certain level of resentment directed towards vaccine refusers. For them, rejecting the vaccine reflected a failure to understand its role in preventing disease and cross-infection. Some participants who showed positive attitudes towards vaccination linked it to the importance of vaccines in keeping them alive during the COVID-19 pandemic or protecting them against diseases. A female participant, in her 50s, from Somkhele, referring to the COVID-19 pandemic, noted:

Yes, yes, I can say that the introduction of COVID-19 was scary, and I was one of the people who became scared… Let me say that I was scared because…they said that it is a pandemic which affects people in faraway places, but when it was here, we got scared because we saw people dying like flies… You will be scared because you will hear some people who were admitted to the hospital due to headache or coughing as usual, blocked nose; you will hear that they died and the doctor diagnosed them with COVID-19 and you will wonder what kind of a disease is this, which has no cure.

It is not strange to hear this sentiment, considering the level of chaos and uncertainty linked to the advent of COVID-19 in South Africa. The Government instituted lockdowns from March 2020 when it declared a National State of Disaster [40, 41]. With these lockdowns came several regulations limiting movements, (public) gatherings and access, even, to medical facilities and grocery stores [39, 41]. These heightened people’s worries since no cure was in sight. Furthermore, news of deaths of government officials (including the Minister in the Presidency, Jackson Mthembu) [50, 51], the Zulu King (King Goodwill Zwelithini) [52], and participants’ observation of deaths in their own communities reinforced these fears. These realities linked with the lack of any proven treatment– except supportive care– would have propelled people to become naturally disposed and ready– and even praying– for the emergence of a vaccine to protect them. These views were prevalent among the older population, potentially reflecting the vulnerability of the elderly, people with terminal illness, and those with other chronic diseases.

### Government campaigns, mandates, penalties and/or incentives

In South Africa, there were major debates regarding whether the government should impose compulsory SARS-CoV-2 vaccination mandates [53, 54]. While government did not establish and enforce such mandates, several institutions, including universities and companies [55, 56] were allowed to institute vaccination policies that granted or denied people a right of access based on vaccination status [53]. Healthcare workers were not required to receive a vaccine, but some employers could, based on an existing legal framework like the National Health Act No. 61 of 2003, apply these mandates, albeit mindful of government’s guidelines [57, 58]. Our study participants highlighted how campaigns championing vaccination, subtle applications of SARS-CoV-2 vaccination mandates and perceived weaponization of penalties and incentives– all contributed to the uptake of vaccines during the pandemic.

A young man from Durban (20–30 years old) noted that ‘campaigns that were held by the government… here in Durban helped a lot to increase the number of people who received the vaccine when comparing provinces'. A woman from Somkhele (40–50 years old) listed the different ways in which participants got information about vaccination campaigns. These included television and radio, healthcare facilities and community caregivers. In addition to these positive campaigns, a participant (20–30 years old) from Durban, and two participants from Somkhele– in their 40s and 80s, respectively– observed that the application of mandates to penalise defaulters emerged in situations in which people were denied access to their university campuses, sporting events, and medical facilities without showing proof of vaccination.

Reducing COVID-19 infections during the pandemic also meant incorporating regular testing in the plan, especially for people who were not getting vaccinated. Participants suggested that the insistence on regular testing and providing proof of a negative test before accessing sensitive locations proved punitive enough for some people to change their minds in favour of vaccination. As some participants noted, ‘people tested for COVID-19; others were forced by the jobs they do to go test’ (Durban participant 20–30 years old), and ‘if you are not vaccinated you must produce proof every Monday that you have tested negative for COVID’ (Somkhele participant 20–30 years old). Another approach was described by a woman (50–60 years old) who ‘…heard on the radio that if you are not vaccinated, you will not be allowed to go to the grocery shops and you will not be allowed to go to many places if you are not vaccinated, that is what I was aware of.’ (This claim was untrue.)

Campaigns were useful to encourage people to embrace vaccination or deepen an understanding among the population about the imperative to vaccinate. Some of the penalties mentioned, real or perceived, appeared to have influenced people’s behaviour. Similarly, some participants suggested that the presence of incentives such as gift cards or vouchers (of 100 and 150 rands [c. USD 9]) proved useful in encouraging people to get vaccinated. A man in his 30s from Somkhele spoke approvingly of the voucher that people received for getting vaccinated.

In all, these initiatives contributed in one way or another towards increasing vaccine uptake among participants .

## Hesitancy: limits to vaccine acceptability

The interviews also highlighted vaccine refusals, and some hesitant views, largely among the younger population. This section highlights some of the issues raised.

### Pluralities

Many participants reported combining biomedical and ‘traditional’ treatments for diseases (medical pluralism). Some advocated for it. Herbs such as leaves from the eucalyptus trees among others were used, with steaming and traditional alternatives, either together with– or as alternatives to biomedicine. A woman (50–60 years old) from Somkhele mentioned traditional therapies or remedies which people believed to be effective for all manner of diseases, including COVID-19:

You know… when the [COVID-19] disease came, people advised us to use a fever tree, and we used it before. Yes, we would take a fever tree, Gumtree, Vicks and water to steam and breathe if we were coughing. I do not want to hide this from you my girl, I did it. I steamed and bathed and gargled because we grew up in times of high fever as I have explained about measles… We used an enema but the most important thing when it comes to this fever was to steam and bath, breathe and you will see that the fever is coming down.

Linked to this are popular beliefs related to diseases, their management, and the lack of full acceptance of biomedicine, something which can be viewed in the context of the wider cultural and medical history of the area. A retired nurse (in her 80s) revealed that:

People generally believe that when there is a disease outbreak they need to first consult with a traditional healer. Their first level of intervention is the traditional healer then the clinic. The traditional healers would then explain that they would give them traditional medicine but their health rests in the person going to vaccinate at the clinic.

Many participants highlighted the existence of a popular belief that people should beat themselves at a particular tree to cure mumps.

Given the long-standing importance of traditional healers within the Zulu society, people and their lineages are often chosen by their ancestors as *Sangomas* (healers) [24]. Their role is crucial in protecting traditional healing practices since they represent their ancestors. Thus, their actions must be in line with ancestral principles. So, people with an ancestral calling (likely to become traditional healers) were deemed to be barred by their ancestors– whom they believe inhabit their bodies– from accepting biomedical therapy, including vaccines. This rule also applies to their children. A female traditional healer (60–70 years old) explained that although she is pro-vaccination, she and her children are not allowed to accept it. She believes that disobeying this command, as she sometimes does, incurs consequences. So, she has to perform sacrifices to ‘placate the ancestors for her disobedience’ [she showed her scars]:

I didn’t have a problem [with vaccination], but my ancestors are against the vaccine; they don’t want it.… I am telling you that my shoulder has been numb since I vaccinated with that vaccine. My back is sore… It is painful… They [the ancestors] want a goat and bile so that I will clean the vaccine. I can’t afford it.… You understand that thing, if I have been vaccinated, my husband needs to slaughter a goat [for my cleansing]… and I need to drink this bile….

This dual approach aimed at managing health and illness is widespread in the Zulu cultural society, and more generally in South Africa, despite advancements in biomedicine. Zulu history and people’s broader social context and networks contribute strongly to this situation, shaping their disposition towards vaccines. We address this social component next.

### Social network influence

#### Religious contexts

Many participants disagreed with the notion that religion and culture– including cultural structures and/or practices– affected engagement with vaccines. Participants, especially the elderly, denied that religion and culture contributed to vaccine hesitancy. They attributed such hesitancy (or outright refusal) to individual idiosyncrasies, fears and ‘stubbornness’. A Roman Catholic man from Somkhele (60–70 years old) noted: ‘No, it [religion or religious denomination] does not prohibit me because the church did not say anything about that, and it does not interfere with the vaccination process. All church members are vaccinated.’ While a man from Somkhele aged between 47 and 52 years said:

I do not think that my traditional practices clash with taking the vaccine, it depends on an individual choice. I think that as a Zulu man, I do not have a problem, even if I analyse it for my benefit. There is nowhere that it is against my tradition, it is just a health issue.

However, there is anecdotal evidence that some Christian denominations discouraged members from accepting vaccines [59]. This was a view supported by one participant, a male from Durban, in his 20s, who reported that members of some religious groups or denominations feared exclusion if they obtained vaccinations:

I doubt religious people are vaccinated because other religions do not agree with Western medicine. They do not do modern things. For instance, the Shembe and Zion, I doubt that they are vaccinated because what can I say, their religion is based a lot on culture. We can say Christianity and all that Roman Catholic are the ones who approve vaccination, but I doubt other religion who are linked to cultures [approve it].

In addition, as explored earlier, people with ancestral calling were also arguably bound by their traditional religious beliefs to avoid biomedicine in both urban and rural contexts. This is in addition to a perceived level of distrust of biomedicine, that is perhaps unsurprising given the historical record, in favour of more indigenous traditional or cultural remedies for diseases and ailments [59].

#### Social media

Many participants highlighted distrust for vaccines because of propaganda shared via social media platforms. An example is one in which a young man from Durban noted that his own father ‘… refused to vaccinate because he used to say that vaccines are the way politicians will use to kill us.’ WhatsApp and Facebook were used to share information, especially among younger people. These stories included religious elements, often linking SARS-CoV-2 vaccines to Satanism (through the biblical mark of the beast– 666). The vaccines were also linked the mobile network 5G, culminating in low levels of insinuations and accusations (emerging from participants) that the South African President, Cyril Ramaphosa, was conspiring with the West to achieve insidious agendas [59].

Similarly, vaccines were also suspected to be risky to one’s fertility, a method of covert sterilisation in line with an alleged depopulation agenda. This created a sense of uncertainty and doubt, especially among the younger participants. A man (20–30 years old) from Durban remarked:

It can happen that everyone who vaccinated will not have a child, you will never know. It will happen that the young males who vaccinated will never reach 40 (years) who knows? You know they said… here in Africa we are close to 5 or 6 billion. Yes the people will be us. So, so they have to depopulate us….

A female nurse (30–40 years old) from Somkhele shared her experience of a similar perspective and misinformation that was pervasive among the population visiting the hospitals. She noted:

They would say when a child gets the vaccine the body temperature rises and sometimes catches the flu. With the COVID vaccine some people were claiming that it causes erectile dysfunction [laughing]; your arm stops working and becomes numb, and you would die after two years… There is a lot.

The preponderance of misinformation transmitted on social media also included health practitioners– not necessarily South African doctors and nurses– who through their comments, body language and attitudes towards vaccines, heightened the level of scepticism already palpable at population levels (during the COVID-19 pandemic). A man from Durban (20–30 years old) noted:

… some videos kept on circulating on social media and these were very disturbing videos because you would find professional doctors, highly known doctors disregarding or disputing vaccine [safety]. So, you know it becomes a problem that doctors are saying this is something that was planned; you understand? So why will I take a vaccine for a planned virus…?

Finally, alleged deaths and side effects linked to SARS-CoV-2 vaccines were also mentioned, causing fear and worry among populations. Participants reported incidents where nurses who had experienced the death of colleagues or patients within the context of SARS-CoV-2 vaccinations shared such information widely, thus raising anxiety within the population. Often, these allegations of vaccine-related deaths were shared simultaneously with those of side-effects or allergic reactions experienced after vaccinations; as a woman, in her 60s, from Somkhele reported, ‘the side effects scared people.’ This meant that the emerging stories shared either through social media platforms or rumours [10, 11], were perceived to increase fear and scepticism among people, contributing to elements of hesitancy, and refusal in extreme cases, within the population. Both younger and older participants appeared to be affected by these stories, although the level of mistrust and consequential impacts was higher among the younger participants.

## COVID-19’s dual effects

Our findings reveal that COVID-19 had a dual effect on vaccine engagement. There was an observed positive consciousness and engagement around vaccines, but also concerns that largely related to high levels of (mis)information and negative propaganda about vaccines. This made more people sceptical and seeking more clarity about vaccines. The questions raised represented a healthy engagement with the subject and greater awareness around the importance of vaccines within their individual and contextual spaces. A young man from Durban observed that:

COVID-19 has taught me not to trust that everything alleged to help me can help me. To not trust that a vaccine can make me have life in abundance but instead know that it can take away from my well-being….

This observation represents some of the more negative outlooks circulating in the public domain and often perpetuated by social media. Government information channels– campaigns and mandates– were useful. However, the preponderance of conflicting information and the manipulation of social media platforms, images and propaganda compounded the problem of hesitancy. This is because they were often led by prominent figures in the context of uncertainties and differing levels of scientific understanding. Indeed, while vaccine uptake among our study participants was high, even adherents were sceptical or hesitant, often hoping that the worst would not happen to them.

An interesting finding within this study, however, is that despite doubts about the SARS-CoV-2 vaccines, some participants’ responses showed their positive inclination to obtain other vaccines. This was especially among the mothers’ continued disposition to take their children for their routine vaccinations. So, the COVID-19 pandemic (or views about the vaccine) did not appear to exert an overt negative impact on many of the study participants’ outlook on other vaccination campaigns.

## Discussion

This study explored how participants living in rural and urban communities in KZN described factors that shape their outlook– and those of others– towards vaccines. Our study was implemented when high-profile debates around vaccine effectiveness, safety, and authenticity, heightened by the COVID-19 pandemic, were centre-stage globally. In South Africa, COVID-19 lockdowns, policies and awareness creation campaigns around the COVID-19 vaccines, and different stories, provided a basis for peoples’ trust and/or scepticism around vaccines. Thus, lessons from this study include the multidimensional factors that shape vaccine perceptions and attitudes.

We document positive attitudes and sentiments towards vaccines and vaccination initiatives. Positive attitudes also cut across age and gender, in line with vaccine studies that show negligible differences between men and women about vaccine acceptability [15, 60, 61]. The majority of our participants maintained relative openness towards vaccination in general, despite some suspicions around SARS-CoV-2 vaccines [15, 59–62]. Although younger participants were more critical of the SARS-CoV-2 vaccine [15, 16, 60]– consistent with established literature– many of them also demonstrated positive attitudes, evidenced by their reported vaccine uptake (Table 1). Interestingly, other studies have found that vaccine hesitancy or distrust for vaccinations seemed to be much higher among older populations, but this was not replicated in our study population [63]. This difference found in other ‘international’ contexts [63], demonstrates the importance of the context-specificity of outlooks on, engagement with, and attitudes toward vaccination. It also further validates our study’s consideration of the importance of localised qualitative work on the subject matter.

Participants' motivations for vaccination included the will to survive, the fear of death and illness, and the need to continue their lives in the society. Similarly highlighted were the place and role of government campaigns, mandates, incentives, and penalties, and peoples’ grasp of the importance of vaccines for diseases and illness prevention, control, survival, and securing the health of family members. These reported drivers resonate with findings from other studies [15, 16, 60]. They also point to broader debate(s) around the centrality of individual choice versus the place of public good wherein getting vaccinated transcends questions of individual rights but emphasises responsibilities to society, their neighbours and the community. It is against this backdrop that some participants, especially older participants, described vaccine-hesitant individuals as `stubborn people’. While this description raises additional questions such as the level or importance of tolerance within the population considering the multidimensional drivers of hesitancy, it still portrays the depth of acceptance of the crucial importance of vaccines [64]. Thus, people who are perceived as wilfully threatening or jeopardising this survival are not often tolerated.

However, even among this largely vaccinated population, positive sentiments do not negate elements of hesitancy, scepticism, fear, and worry. Questions remained about the COVID-19 vaccine alongside beliefs about the importance of alternative or complementary local and/or traditional approaches to health and illness [64–66]. Questions around vaccine safety, effectiveness and efficacy remained evident even among participants who reported accepting vaccination. Indeed, the problem of vaccine confidence transcends the COVID-19 context globally, and it is well captured in the literature [2, 3, 7, 16, 60, 67, 68]. This resonates with studies, including those emphasising parental acceptance or hesitancy, about childhood vaccinations [3, 65, 69]. Parents who refused or delayed vaccinating their children, or chose a different schedule, often presented questions about the safety, effectiveness, and necessity of the vaccines for their newborns [64]. These questions around vaccine confidence thus transcend a simple delineation of people into pro-vaccination and anti-vaccination because perspectives on and attitudes towards vaccines and vaccinations can be nuanced, sometimes ambivalent, changing and dynamic across people, time and different vaccines [61, 64–66, 69, 70].

In our study, many participants did not recognise religion and cultural belief systems as powerful drivers of vaccine engagement, including hesitancy. However, these factors did contribute to how some people engaged with vaccines. The extensive literature on medical pluralism confirms this– explaining how the cultural and religious inclination of (South) African populations affect their engagement with biomedicine [24, 26]. This dual engagement is recorded where people rely on ‘traditional’ healers and ‘traditional’ medicine to address their psycho-spiritual needs while biomedicine serves their more physical health needs [19–21, 23, 24]. In some cases, these are mutually exclusive, while in some others, people merge these views, as seen in our study. This even applies in the case of individuals ‘called’ to be traditional healers– *Izangoma*. Their standing as representatives of their ancestors can, to a great extent, shape their engagement with biomedicine [59].

Participants’ views about perceived fear of exclusion from some religious groups, the fear of ancestors’ reactions, and the sentiments about the mark of the beast or initiation to satanism were described as shaping attitudes towards vaccines [59]. Social contexts shape vaccine perceptions and engagement and, in the context of childhood immunisation, ‘… parents are often communicating not just what they think about vaccines, but also who they are, what they value, and with whom they identify’ [15, 16]. Indeed, the social worlds in which people exist often affect the way they perceive and engage with these vaccines, hence the importance of understanding the various impacts of religion and culture.

In the South African context, the spread of (mis)information and propaganda further underpinned sentiments that portrayed hesitancy, even among those fully vaccinated. However, most hesitancy that was voiced was about SARS-CoV-2 vaccines, and largely among younger age groups, which is also well captured in the literature [16].

One threat of the preponderance of (mis)information about the SARS-CoV-2 vaccine through social media concerns its potential impact– real or imagined– on how people engage with or perceive vaccines in general. Vaccine hesitancy can be fuelled by fears, stories, or experiences of side effects– including myths linking specific vaccines to autism and other adverse events– as well as concerns about possible collusion between government and pharmaceutical companies [5, 7, 64, 65, 69–71]. Under such circumstances, especially in cases of childhood vaccinations, hesitancy transcends a simple refusal or decision to delay [64]. Instead, parents’ protective instincts force them to choose actions that they perceive to be less likely to inflict irreparable damage on their children, irrespective of what vaccination guidelines recommend [64]. Social media platforms have become an arena for the dissemination of varying thematic issues (and protests) related to the COVID-19 pandemic: from mask-wearing to vaccine mandates [72]. Such debates, protests, and sometimes, the outright use of conspiracy theories and cyberbullying, can fuel hesitancy [72].

The avalanche of (mis)information on social media, and insufficient high quality vaccination messaging, can impact vaccine engagement. Although our study suggests that most participants did not allow such information to dissuade them from accepting other vaccines or allowing their children to be vaccinated, there is literature to suggest that the COVID-19 pandemic may have impacted broad-level engagements with general vaccines in South Africa– and beyond [15, 16, 60]. Drawing on a review of seven surveys regarding vaccine perceptions in South Africa, the negative externalities of the COVID-19 pandemic may be an exacerbation of ‘current vaccine hesitancy in the country’ because some of these surveys showed that ‘belief in the serious health side effects of vaccines and preference for infection-acquired immunity… increased significantly since the COVID-19 national lockdowns…’ [15].

## Study limitations

Our study has some limitations, mainly related to its conduct during the COVID-19 pandemic and associated lockdown regulations. We therefore had to rely partly on convenience sampling, incorporated with other techniques. We had many no-shows as some potential participants, sourced through our networks and snowball technique, declined to participate, forcing us to rely on those available. There is a risk that those who were anti-vaccine or had remained unvaccinated were less likely to participate than their vaccinated counterparts, despite efforts to recruit them to balance the perspectives. Consequently, our survey potentially over-represents positive views and under-represents hesitancy. However, as a result of living within the same society, these participants can nevertheless provide insights into pervasive anti-vaccine rhetoric within their communities.

## Recommendations and conclusion

Our research showed that vaccine perception and engagement in KZN (South Africa) is complex: the outlooks and attitudes described here are diverse and heterogeneous. Our findings show that exposures to social media, cultural and religious influences, and daily interactions, influence how people understand and perceive vaccines. Participants’ viewpoints demonstrated the complexities surrounding the concept or idea of vaccine hesitancy; these perspectives are crucial for a better and deeper understanding of vaccine confidence and acceptance in South Africa and the factors that drive it. The growing importance of social media as an influential driver of vaccine hesitancy also emerged, even for people not directly exposed to it (like some older people), but who are told by others about messages shared via social media. Equally important is the place of medical pluralism in people’s engagement with health interventions such as vaccine-related interventions. It follows that exploring the drivers of vaccine hesitancy, over time and in communities occupying different geographical and cultural spaces, provides opportunities to understand why vaccines are accepted or rejected.

The study findings also point to an imperative for effective community engagement and robust awareness-creation around vaccines. This also entails paying attention to the multi-dimensionalities and complexities of populations’ understanding and engagement with biomedicine in their local context. Community engagement tailored to local concerns, effective information sharing and awareness creation around vaccines and vaccination initiatives will benefit vaccine-related planning and interventions for people of different cultural, religious, and intellectual leanings. The issue of vaccine hesitancy is more complex than people refusing vaccines. Deeper engagement, drawing in perspectives from across the humanities and social sciences, is therefore crucial to optimising vaccine interventions.

## Data Availability

All the data generated and/or analysed for this study and the ensuing manuscript are not publicly available in order to maintain the privacy and confidentiality component of the study informed consent agreements made with study participants. AHRI maintains a data repository for all data generated under this study and this can be made available upon request.

## Abbreviations

AHRI: Africa Health Research Institute
CAB: Community Advisory Board
COVID-19: Coronavirus Disease 2019
KZN: KwaZulu-Natal Province
PED: Public Engagement Department
WHO: World Health Organization
SARS-CoV-2: Severe Acute Respiratory Syndrome Coronavirus 2

## References

[1] Ten threats to global health in. 2019 [https://www.who.int/news-room/spotlight/ten-threats-to-global-health-in-2019]

[2] Cooper S, Betsch C, Sambala EZ, McHiza N, Wiysonge CS. Vaccine hesitancy– a potential threat to the achievements of vaccination programmes in Africa. Hum Vaccines Immunotherapeutics. 2018;14(10):2355–7. 10.1080/21645515.2018.1460987 PMC6284499 29617173

[3] Peretti-Watel P, Larson HJ, Ward JK, Schulz WS, Verger P. Vaccine hesitancy: clarifying a theoretical framework for an ambiguous notion. PLoS Curr 2015, 7. 10.1371/currents.outbreaks.6844c80ff9f5b273f34c91f71b7fc289 PMC4353679 25789201

[4] Rosselli R, Martini M, Bragazzi NL. The old and the new: vaccine hesitancy in the era of the web 2.0. Challenges and opportunities. J Prev Med Hyg. 2016;57(1):E47–50. 27346940 PMC4910443

[5] Paterson P, Meurice F, Stanberry LR, Glismann S, Rosenthal SL, Larson HJ. Vaccine hesitancy and healthcare providers. Vaccine. 2016;34(52):6700–6. 27810314 10.1016/j.vaccine.2016.10.042

[6] MacDonald NE. Vaccine hesitancy: definition, scope and determinants. Vaccine. 2015;33(34):4161–4. 25896383 10.1016/j.vaccine.2015.04.036

[7] Larson HJ, Jarrett C, Eckersberger E, Smith DM, Paterson P. Understanding vaccine hesitancy around vaccines and vaccination from a global perspective: a systematic review of published literature, 2007–2012. Vaccine. 2014;32(19):2150–9. 24598724 10.1016/j.vaccine.2014.01.081

[8] Kumar D, Chandra R, Mathur M, Samdariya S, Kapoor N. Vaccine hesitancy: Understanding better to address better. Isr J Health Policy Res. 2016;5(1):2. 26839681 10.1186/s13584-016-0062-y PMC4736490

[9] World Health Organisation. Report of the SAGE working group on vaccine hesitancy. In.: World Health Organisation; 2014.

[10] Feldman-Savelsberg P, Ndonko FT, Schmidt-Ehry B. Sterilizing vaccines or the politics of the womb: retrospective study of a rumor in Cameroon. 2000, 14(2):159–79. 10.1525/maq.2000.14.2.159 10879368

[11] Kaler A. Health interventions and the persistence of rumour: the circulation of sterility stories in African public health campaigns. Soc Sci Med. 2009;68(9):1711–9. 19282080 10.1016/j.socscimed.2009.01.038

[12] Renne E. Perspectives on polio and immunization in Northern Nigeria. Soc Sci Med. 2006;63(7):1857–69. 16765498 10.1016/j.socscimed.2006.04.025

[13] Olufowote JO, Livingston DJ. The excluded voices from Africa’s Sahel: alternative meanings of health in narratives of resistance to the global polio eradication initiative in Northern Nigeria. Health Commun. 2022;37(11):1389–400. 33685303 10.1080/10410236.2021.1895416

[14] Ghinai I, Willott C, Dadari I, Larson HJ. Listening to the rumours: what the Northern Nigeria polio vaccine boycott can tell Us ten years on. Glob Public Health. 2013;8(10):1138–50. 24294986 10.1080/17441692.2013.859720 PMC4098042

[15] Cooper S, van Rooyen H, Wiysonge CS. COVID-19 vaccine hesitancy in South Africa: how can we maximize uptake of COVID-19 vaccines? Expert Rev Vaccines. 2021;20(8):921–33. 34252336 10.1080/14760584.2021.1949291

[16] Cooper S, Van Rooyen H, Wiysonge CS. COVID-19 vaccine hesitancy in South Africa: A complex social phenomenon. South Afr Med J = Suid-Afrikaanse Tydskrif vir Geneeskunde. 2021;111(8):702–3. 35227348 10.7196/SAMJ.2021.v111i8.15800

[17] Flint K. Africa isn’t a testing lab: considering COVID vaccine trials in a history of biomedical experimentation and abuse. J West Afr History. 2020;6(2):126–40.

[18] Noble V. Anne Digby, Diversity and division in medicine: health care in South Africa from the 1800s, Studies in the History of Medicine, vol. 5, Oxford, Peter Lang, 2006, p. 504, illus., £49.50 (paperback 978-0-8204-7978-0). *Medical History* 2008, 52(4):550–551.

[19] Khalid A. Reviewed works: plural medicine, tradition and modernity, 1800–2000 by Waltraud Ernst; healing traditions: African medicine, cultural exchange, and competition in South Africa by Karen E. Flint. Review by: Amna Khalid. Kronos. 2010;36:320–5.

[20] Flint KE. Healing traditions: African medicine, cultural exchange, and competition in South Africa, 1820–1948. Athens: Ohio University Press; 2008.

[21] Bakshi P: Reviewed Work: Healing Traditions: African Medicine, Cultural Exchange, and Competition in South Africa, 1820–1948 by, Karen E, Flint. Reviewed by Preston Bakshi. *The Business History Review* 2009, 83(4):867–869.

[22] Bannister P. Regulating ‘tradition’ South Africa Izangoma and the traditional health practitioners act 2004. Camb Anthropol. 2007;27(1):25–61.

[23] Thornton R. The transmission of knowledge in South African traditional healing. Africa: J Int Afr Inst. 2009;79(1):17–34.

[24] King B. We pray at the church in the day and visit the Sangomas at night: health discourses and traditional medicine in rural South Africa. Ann Assoc Am Geogr. 2012;102(5):1173–81.

[25] Kale R. Traditional healers in South Africa: A parallel health care system. BMJ: Br Med J. 1995;310(6988):1182–5. 7767156 10.1136/bmj.310.6988.1182 PMC2549561

[26] Moshabela M, Zuma T, Gaede B. Bridging the gap between biomedical and traditional health practitioners in South Africa. South Afr Health Rev. 2016;2016(1):83–92.

[27] Feierman S. Struggles for control: the social roots of health and healing in modern Africa. Afr Stud Rev. 1985;28(2/3):73–147. 11616461

[28] Union of South Africa: Annual report of the Department of Public Health /. Union of South Africa. In. Edited by Department of Health. Pretoria: Government Printing and Stationery Office; 1929.

[29] Union of South Africa: Annual report of the Department of Public Health /. Union of South Africa. In. Edited by Department of Health. Pretoria: Government Printing and Stationery Office; 1925.

[30] Public Health Department. Medical Officer’s annual report [to] Durban Corporation. In. Durban (South Africa): The Corporation; 1972.

[31] South African Medical and Dental Council. The 1928 medical, dental and pharmacy act. In. Cape Town: Cape Times; 1948.

[32] Ngubane H. Aspects of clinical practice and traditional organization of Indigenous healers in South Africa. Social Sci Med Part B: Med Anthropol. 1981;15(3):361–5. 10.1016/0160-7987(81)90061-2 7313722

[33] Mawere M, Awuah-Nyamekye S. Between rhetoric and reality: the state and use of Indigenous knowledge in post-colonial Africa. 1st ed. Cameroon: African Books Collective; 2015.

[34] AHRI - About Us. [https://www.ahri.org/about/]

[35] eThekwini. Municipality– About us (eThekwini Municipality) [https://www.durban.gov.za/pages/government/about-ethekwini]

[36] Zuma T, Seeley J, Hlongwane S, Chimbindi N, Sherr L, Floyd S, Birdthistle I, Shahmanesh M. A socio-ecological approach to Understanding experiences and perceptions of a multilevel HIV prevention intervention: the determined, resilient, empowered, AIDS-free, mentored, and safe (DREAMS) partnership in uMkhanyakude, KwaZulu-Natal, South Africa. SSM - Qualitative Res Health. 2022;2:100138.

[37] Gareta D, Baisley K, Mngomezulu T, Smit T, Khoza T, Nxumalo S, Dreyer J, Dube S, Majozi N, Ording-Jesperson G, et al. Cohort profile update: Africa centre demographic information system (ACDIS) and population-based HIV survey. Int J Epidemiol. 2021;50(1):33–4. 33437994 10.1093/ije/dyaa264 PMC7938501

[38] Orievulu K, Ayeb-Karlsson S, Ngwenya N, Ngema S, McGregor H, Adeagbo O, Siedner MJ, Hanekom W, Kniveton D, Seeley J, et al. Economic, social and demographic impacts of drought on treatment adherence among people living with HIV in rural South Africa: A qualitative analysis. Clim Risk Manage. 2022;36:100423. 10.1016/j.crm.2022.100423 PMC7614312 36923966

[39] Republic of South Africa: Disaster Management Act. 2002: Amendment of Regulations issued in terms of Sect. 27(2) - Gazette No: 43168. In. Online: Department of Co-operative Governance and Traditional Affairs; 2020.

[40] Republic of South Africa: Disaster Management Act. 2002: Declaration of a National State of Disaster (Gazette No: 43096). In. Online: Department of Co-operative Governance and Traditional Affairs; 2020.

[41] Republic of South Africa: Disaster Management Act. 2002: Regulations Issued in terms of Sect. 27(2) of the Disaster Management Act, 2002 (Gazette No: 43107). In. Online: Department of Co-operative Governance and Traditional Affairs; 2020.

[42] Republic of South Africa. South Africa: Matrix of COVID-19 Related Regulations and Measures. In. Edited by Department of Planning MaE. Online: Department of Planning, Monitoring and Evaluation; 2021.

[43] Suri H. Purposeful sampling in qualitative research synthesis. Qualitative Res J. 2011;11(2):63–75.

[44] Robinson OC. Sampling in Interview-Based qualitative research: A theoretical and practical guide. Qualitative Res Psychol. 2014;11(1):25–41.

[45] Turner DP. Sampling methods in research design. Headache: J Head Face Pain 2020, 60(1). 10.1111/head.13707 31913516

[46] Kiger ME, Varpio L. Thematic analysis of qualitative data: AMEE guide 131. Med Teach. 2020;42(8):846–54. 32356468 10.1080/0142159X.2020.1755030

[47] Clarke V, Braun V. Thematic analysis. J Posit Psychol. 2017;12(3):297–8.

[48] Braun V, Clarke V. Using thematic analysis in psychology. Qualitative Res Psychol. 2006;3(2):77–101.

[49] Ashkir S, Khaliq OP, Hunter M, Moodley J. Maternal vaccination: A narrative review. South Afr J Infect Dis. 2022;37(1):451. 10.4102/sajid.v37i1.451 PMC9575370 36262428

[50] News24 Politics Team: Jackson Mthembu dies of Covid-19-related complications. In. Online. 2021: https://www.news24.com/news24/southafrica/news/jackson-mthembu-dies-of-covid-19-related-complications-20210121-20210122

[51] Hauser J, Swails B. South African government minister Jackson Mthembu dies of Covid-19 In: *CNN.* Online: CNN World; 2021.

[52] Makhafola G. King Goodwill Zwelithini died of Covid-19-related complications - Buthelezi. new24 online. Online; 2021.

[53] Moodley K. Vaccine mandates in South Africa: where are they most needed? The conversation. Online: The conversation Africa, Inc.; 2021.

[54] Moodley K. The ethics behind mandatory COVID-19 vaccination post-Omicron: the South African context. South Afr J Sci 2022, 118(5/6).

[55] Karim SA. Covid vaccine mandates don’t have to undermine your rights. WITS news. Online: WITS University; 2021.

[56] Mthembu X. Here are some of the country’s companies who have announced mandatory Covid-19 vaccine policies for their employees. In: *IOL.* Online: IOL; 2021.

[57] van Vuuren CJJ, van Vuuren JMJ. Perspectives of healthcare workers in South Africa on COVID-19 vaccination passports. Health SA = SA Gesondheid. 2022;27:1823. 35548060 10.4102/hsag.v27i0.1823 PMC9082080

[58] Employment and Labour Minister issues new. direction with regard to vaccination inthe workplace [https://www.labour.gov.za/employment-and-labour-minister-issues-new-direction-with-regard-to-vaccination-in-the-workplace#:~:text=The%20Consolidated%20OHS%20Direction%20now,operational%20requirements%20of%20the%20workplace.].

[59] Netshapapame TS. COVID-19 vaccination hesitancy in South Africa: biblical discourse. HTS: Theological Stud. 2023;79(4):7795.

[60] Katoto P, Parker S, Coulson N, Pillay N, Cooper S, Jaca A, Mavundza E, Houston G, Groenewald C, Essack Z et al. Predictors of COVID-19 vaccine hesitancy in South African local communities: the VaxScenes study. Vaccines 2022, 10(3). 10.3390/vaccines10030353 PMC8951818 35334991

[61] Engelbrecht M, Heunis C, Kigozi G. COVID-19 vaccine hesitancy in South Africa: lessons for future pandemics. Int J Environ Res Public Health 2022, 19(11). 10.3390/ijerph19116694 PMC9180246 35682278

[62] Bardosh K, de Figueiredo A, Gur-Arie R, Jamrozik E, Doidge J, Lemmens T, Keshavjee S, Graham JE, Baral S. The unintended consequences of COVID-19 vaccine policy: why mandates, passports and restrictions May cause more harm than good. BMJ Global Health 2022, 7(5). 10.1136/bmjgh-2022-008684 PMC9136690 35618306

[63] Tan M, Straughan PT, Cheong G. Information trust and COVID-19 vaccine hesitancy amongst middle-aged and older adults in Singapore: A latent class analysis Approach. *Social science & medicine (1982)* 2022, 296:114767. 10.1016/j.socscimed.2022.114767 PMC8812088 35144226

[64] Dubé E, Vivion M, Sauvageau C, Gagneur A, Gagnon R, Guay M. Nature does things well, why should we interfere?? Vaccine hesitancy among mothers. Qual Health Res. 2016;26(3):411–25. 25711847 10.1177/1049732315573207

[65] Williams SE. What are the factors that contribute to parental vaccine-hesitancy and what can we do about it? Hum Vaccin Immunother. 2014;10(9):2584–96. 25483505 10.4161/hv.28596 PMC4977453

[66] Dubé E, Laberge C, Guay M, Bramadat P, Roy R, Bettinger JA. Vaccine hesitancy. Hum Vaccines Immunotherapeutics. 2013;9(8):1763–73. 10.4161/hv.24657 PMC3906279 23584253

[67] Cooper S, Schmidt BM, Sambala EZ, Swartz A, Colvin CJ, Leon N, Betsch C, Wiysonge CS. Factors that influence parents’ and informal caregivers’ acceptance of routine childhood vaccination: a qualitative evidence synthesis. Cochrane Database Syst Rev 2019, 2019(2). 10.1002/14651858.CD013265.pub2 PMC8550333 34706066

[68] Cooper S, Schmidt BM, Ryan J, Leon N, Mavundza E, Burnett R, Tanywe AC, Wiysonge CS. Factors that influence acceptance of human papillomavirus (HPV) vaccination for adolescents: a qualitative evidence synthesis. Cochrane Database Syst Rev 2019, 2019(9). 10.1002/14651858.CD013430.pub2 PMC11998976 40232221

[69] Wang E, Baras Y, Buttenheim AM. Everybody just wants to do what’s best for their child: Understanding how pro-vaccine parents can support a culture of vaccine hesitancy. Vaccine. 2015;33(48):6703–9. 26518397 10.1016/j.vaccine.2015.10.090 PMC5554443

[70] Bedford H, Attwell K, Danchin M, Marshall H, Corben P, Leask J. Vaccine hesitancy, refusal and access barriers: the need for clarity in terminology. Vaccine. 2018;36(44):6556–8. 28830694 10.1016/j.vaccine.2017.08.004

[71] Dubé E. Addressing vaccine hesitancy: the crucial role of healthcare providers. Clin Microbiol Infect. 2017;23(5):279–80. 27851999 10.1016/j.cmi.2016.11.007

[72] Martin S, Vanderslott S. Any Idea how fast ‘it’s just a mask!’ can turn into ‘it’s just a vaccine!’: from mask mandates to vaccine mandates during the COVID-19 pandemic. Vaccine. 2022;40(51):7488–99. 34823912 10.1016/j.vaccine.2021.10.031 PMC8552554

